# Heterogeneous mechanisms, risk stratification, and translational advances in the malignant transformation of oral potentially malignant disorders to oral squamous cell carcinoma

**DOI:** 10.3389/fmolb.2026.1948915

**Published:** 2026-09-18

**Authors:** Zijing Yu, Hutao Peng, Xiaobo Cheng, Wenpin Xu, Yupei Wang, Yinhu Xiao, Ting Wei, Zhizhen Huang, Yang He, Jian Wang

**Affiliations:** 1 Department of Stomatology, Taihe Hospital, Hubei University of Medicine, Shiyan, Hubei, China; 2 Institute of Oral Diseases, School of Dentistry, Hubei University of Medicine, Shiyan, Hubei, China

**Keywords:** oral erythroplakia, oral leukoplakia, oral lichen planus, oral potentially malignant disorders, oral squamous cell carcinoma, oral submucous fibrosis, precision prevention, proliferative verrucous leukoplakia

## Abstract

Oral potentially malignant disorders (OPMDs) represent important precursor conditions in the development of oral squamous cell carcinoma (OSCC) and provide a critical window for early prevention, risk assessment, and precision intervention of oral cancer. Oral leukoplakia, proliferative verrucous leukoplakia, oral erythroplakia, oral lichen planus, and oral submucous fibrosis are among the most common and clinically significant OPMDs. Although these disorders may contribute to malignant transformation through shared mechanisms, including chronic inflammation, oxidative stress, genetic and epigenetic alterations, immune dysregulation, epithelial–mesenchymal crosstalk, and microbiome dysbiosis, their predominant pathological processes and malignant transformation trajectories exhibit substantial heterogeneity. Oral leukoplakia is characterized by marked clinical and molecular heterogeneity, genomic instability, and field cancerization effects; Proliferative verrucous leukoplakia is characterized by progressive multifocal disease, an exceptionally high risk of malignant transformation, and a pronounced propensity to develop multiple primary oral cancers; oral erythroplakia is frequently associated with high-grade epithelial dysplasia or early invasive carcinoma; oral lichen planus represents an immune-mediated chronic inflammatory microenvironment that may facilitate tumorigenesis; and oral submucous fibrosis represents a matrix-driven precancerous condition primarily characterized by areca nut exposure and fibrotic extracellular matrix remodeling. In recent years, advances in single-cell omics, spatial omics, salivary liquid biopsy, digital pathology, artificial intelligence, and local drug delivery systems have provided emerging technological platforms for dynamic risk stratification, early diagnosis, and precision prevention of malignant transformation in OPMDs. This review summarizes the epidemiological links between major OPMDs and OSCC, disease-specific and shared mechanisms underlying malignant transformation, candidate biomarkers, risk stratification strategies, and advances in translational therapies. Furthermore, it aims to establish an integrated management framework for oral potentially malignant lesions based on the “epithelial–immune–stromal–microbial” niche.

## 1 Introduction

OSCC is one of the most common oral malignancies. Despite substantial advances in surgery, radiotherapy, chemotherapy, targeted therapy, and immunotherapy, the overall prognosis of OSCC remains unsatisfactory, with local recurrence, cervical lymph node metastasis, and second primary tumors representing major factors affecting patients’ long-term survival (Du et al., 2020; Wu et al., 2025; Global Burden of Disease, 2019 Cancer Collaboration et al., 2022; Bray et al., 2024; Lee et al., 2021; Tan et al., 2023). Compared with reactive treatment after the establishment of invasive carcinoma, identifying high-risk lesions and implementing effective interventions during the precancerous stage may provide greater potential to reduce OSCC incidence and mortality. Therefore, OPMDs provide a critical opportunity for both primary and secondary prevention of OSCC (Warnakulasuriya et al., 2021).

Conventional OPMD management primarily relies on clinical features and histopathological grading of epithelial dysplasia; however, this approach has substantial limitations (Warnakulasuriya, 2019). Importantly, some lesions with mild dysplasia may undergo malignant transformation, whereas others with moderate-to-severe dysplasia may remain stable during long-term follow-up (Chaturvedi et al., 2020; Pimenta-Barros et al., 2025). Moreover, substantial clinical, biological, and prognostic heterogeneity exists even within individual disease entities. Therefore, OPMDs should not be regarded simply as “oral precancerous plaques,” but rather as a heterogeneous disease spectrum shaped by diverse risk exposures, immune states, stromal microenvironments, genetic backgrounds, and microbial ecosystems.

Recent studies have highlighted that OPMDs with similar clinical phenotypes may harbor substantially different molecular landscapes and malignant transformation potentials, underscoring the necessity of molecular-based risk stratification (Li et al., 2021; Kumari et al., 2022). In this context, redefining the continuum of malignant transformation from OPMDs to OSCC and establishing multidimensional dynamic risk stratification systems have become major priorities in the management of oral potentially malignant disorders. Accordingly, this review adopts a disease-oriented comparative framework to examine major OPMDs across several interrelated dimensions, including clinical features and patterns of malignant transformation, major disease-specific mechanisms, molecular and microenvironmental alterations, potential biomarkers, and their relevance to risk stratification and clinical management. Rather than treating OPMDs as a homogeneous group of lesions, this framework aims to distinguish disease-specific progression pathways from shared mechanisms underlying malignant transformation and to link these differences to clinically relevant approaches to risk assessment and management (see Table 1).

**TABLE 1:** Comparative characteristics and malignant transformation patterns of major oral potentially malignant disorders.

OPMD	Representative clinical/evolutionary features	Malignant transformation pattern	Predominant disease-specific mechanisms	Clinical implications	Representative references
OLK	Heterogeneous presentation; focal or multifocal lesions; variable epithelial dysplasia	Variable malignant potential; transformation may occur despite low-grade histopathological changes	Genomic instability; TP53/CDKN2A alterations; LOH/CNA accumulation; immune suppression; field cancerization	Integrate clinical, histopathological, and molecular features rather than relying solely on dysplasia grade	(Chaturvedi et al., 2020; Pimenta-Barros et al., 2025; Bhattarai et al., 2024; Wils et al., 2023; Normando et al., 2023; Cai et al., 2023; Zhang et al., 2024a; Gates et al., 2025)
PVL	Persistent, progressive, multifocal disease; recurrent lesions and emergence at new oral sites	Exceptionally high, sustained malignant potential; frequent recurrence and multiple primary oral cancers	Field cancerization; immune dysregulation; transcriptomic and epigenetic remodeling	Long-term whole-mouth surveillance; longitudinal evolution is more informative than single-biopsy dysplasia grade	(Warnakulasuriya et al., 2021; Thompson et al., 2021; Palaia et al., 2021; Fernandes et al., 2023; Farah et al., 2024; Herreros-Pomares et al., 2021; Zhang et al., 2024b)
OE	Persistent red or red-white lesions; often associated with marked epithelial atypia or high-grade dysplasia	High malignant potential with relatively rapid progression	Early TP53 pathway disruption and stem cell-associated epithelial phenotypes, often in a high-grade dysplastic background	Timely biopsy with adequate sampling; excision or close surveillance guided by histopathology	(Villa et al., 2025; Das et al., 2022; Shafer and Waldron, 1975; Lorenzo-Pouso et al., 2022; Qin et al., 1999)
OLP	Chronic immune-inflammatory mucosal disease; erosive, atrophic, erythematous, or reticular phenotypes	Relatively low overall malignant potential, concentrated in selected clinical and biological phenotypes	Persistent T-cell-mediated epithelial injury; chronic inflammation; immune dysregulation; epithelial-immune-stromal interactions	Long-term surveillance for persistent, erosive/atrophic, erythematous, or tongue lesions; risk is heterogeneous	(Louisy et al., 2024; González-Moles et al., 2019; Ramos-García et al., 2021; González-Moles et al., 2022; El-Howati et al., 2023; Maciel et al., 2025; Keim-Del Pino et al., 2024; Wang et al., 2024)
OSMF	Progressive mucosal fibrosis, fibrous bands, and restricted mouth opening associated with areca nut exposure	Malignant potential linked to chronic fibrotic remodeling, with substantial interindividual variability	TGF-β/SMAD activation; oxidative stress; hypoxia; ECM remodeling; matrix stiffening; epithelial-stromal interactions	Areca nut cessation; monitoring of fibrosis/trismus; long-term surveillance	(Tang et al., 2025; Hu et al., 2026a; Xiong et al., 2025; Xu et al., 2024a; Tilakaratne et al., 2006; Li et al., 2023b)

### 1.1 Search strategy and selection criteria

A literature search was conducted in PubMed from March 25 to 23 May 2026, using the terms “oral leukoplakia” or “leukoplakia,” “proliferative verrucous leukoplakia,” “erythroplakia,” “oral lichen planus” or “lichen planus, oral,” “oral submucous fibrosis,” “oral potentially malignant disorders,” and “oral squamous cell carcinoma,” in combination with terms related to malignant transformation, molecular mechanisms, biomarkers, risk stratification, and treatment. Priority was given to studies published within the preceding 5 years. Earlier studies were also included when they provided important original evidence or when recent evidence was limited, particularly for oral erythroplakia. Studies were selected based on their relevance to malignant transformation, underlying mechanisms, biomarkers, risk stratification, and clinical management of oral potentially malignant disorders.

## 2 Epidemiological associations between major OPMDs and OSCC

### 2.1 Oral leukoplakia

Oral leukoplakia (OLK) is the most common OPMD and exhibits marked clinical and molecular heterogeneity. The risk of malignant transformation is influenced by multiple factors, including sex, lesion location, size, multifocality, non-homogeneous or verrucous morphology, epithelial dysplasia, and exposure to carcinogenic factors such as tobacco, alcohol, and areca nut (Pimenta-Barros et al., 2025; Zhu et al., 2021; Bhattarai et al., 2024). Lesions involving the lateral tongue, ventral tongue, and floor of the mouth are generally considered to carry a higher risk of malignant transformation (Pimenta-Barros et al., 2025). A large retrospective cohort study using electronic health records reported an 8.4% malignant transformation rate for OLK, with a median time to malignant transformation of approximately 25 months; HIV-positive status was associated with an increased risk of malignant transformation (Villa et al., 2025).

### 2.2 Proliferative verrucous leukoplakia

Proliferative verrucous leukoplakia (PVL) is recognized as a distinct oral potentially malignant disorder rather than a subtype of conventional oral leukoplakia (Warnakulasuriya et al., 2021). PVL typically follows a persistent and progressive clinical course, initially presenting as one or more white keratotic lesions that gradually enlarge and extend to involve additional oral sites; with disease progression, the lesions may become verrucous or exophytic (Warnakulasuriya et al., 2021). Because histopathologic alterations in early lesions may be subtle, establishing a definitive diagnosis of PVL requires longitudinal clinicopathologic assessment, particularly to determine whether the lesions become multifocal or show progressive enlargement (Thompson et al., 2021).

PVL is characterized by an exceptionally high and sustained risk of malignant transformation. A meta-analysis of 22 studies involving 699 patients reported a malignant transformation rate of 45.8% over a mean follow-up of 7.2 years, with a mean interval of approximately 4.1 years from diagnosis to malignant transformation. Among patients who developed malignancy, 46.5% developed multiple primary oral cancers at distinct oral sites (Palaia et al., 2021). PVL occurs predominantly in middle-aged and older individuals, with a higher prevalence among women, whereas traditional tobacco and alcohol exposure is relatively uncommon. The gingiva and alveolar ridge are commonly involved and also represent frequent sites of malignant transformation (Palaia et al., 2021). Its progressive multifocal course, high recurrence rate, and propensity for multiple primary cancers collectively indicate that PVL represents a high-risk OPMD with distinctive clinical characteristics.

### 2.3 Oral erythroplakia

Oral erythroplakia (OE) is a relatively uncommon OPMD with a very high risk of malignant transformation, clinically presenting as persistent intensely red or velvety plaques that cannot be scraped off, with some lesions containing white keratotic components (Das et al., 2022). The characteristic red appearance of OE is associated with epithelial atrophy, reduced keratinization, prominent visualization of vessels within the lamina propria, and inflammatory infiltration, and is frequently accompanied by marked cytological atypia and impaired epithelial maturation (Shafer and Waldron, 1975). The histopathological spectrum of OE ranges from varying degrees of epithelial dysplasia and carcinoma *in situ* to early-stage squamous cell carcinoma; therefore, a high index of clinical suspicion is warranted upon initial detection (Neville and Day, 2002). Studies have reported that the overall malignant transformation rate of OE ranges from 19.9% to 45%, with some large electronic health record cohorts reporting rates as high as 50.0%. The median time to malignant transformation is only 3.7 months, highlighting its rapid malignant evolution and high-risk nature (Villa et al., 2025; Lorenzo-Pouso et al., 2022; Iocca et al., 2020; Villa et al., 2011; Öhman et al., 2023). Clinically, persistent unexplained red or red-and-white lesions should be biopsied promptly. When necessary, multiple-site sampling or complete excision should be considered, followed by long-term surveillance.

### 2.4 Oral lichen planus

Oral lichen planus (OLP) is a T cell-mediated chronic immune-inflammatory mucosal disorder, and its malignant transformation risk remains controversial. This controversy mainly arises from variations in diagnostic criteria, inconsistent distinction between OLP and oral lichenoid lesions (OLLs) as separate disease entities, differences in follow-up duration, and the presence of coexisting risk exposures (Louisy et al., 2024). Recent studies suggest that the overall malignant transformation rate of OLP is approximately 1%–2%, whereas oral lichenoid lesions may exhibit a higher risk of malignant transformation (González-Moles et al., 2019; Ramos-García et al., 2021; González-Moles et al., 2022). Large-scale genetic and epidemiological studies have demonstrated that, after excluding concomitant leukoplakia, patients with OLP still have significantly increased risks of tongue, gingival, and lip cancers, with an approximately 1.9% 10-year risk of oral cancer development (Re et al., 2024). Therefore, the malignant transformation risk of OLP should not be overestimated; however, closer surveillance is recommended for patients with tongue involvement, erosive or atrophic forms, erythematous lesions, and persistent inflammatory activity.

### 2.5 Oral submucous fibrosis

Oral submucous fibrosis (OSMF) is strongly associated with areca nut chewing and represents a substantial health burden, particularly among populations in South Asia, Southeast Asia, and associated immigrant communities (Reichart and Nguyen, 2008; Gupta and Jawanda, 2021; Cirillo et al., 2022; Tang et al., 2025). Its clinical manifestations include burning pain, mucosal pallor, fibrous band formation, and progressive restriction of mouth opening (Warnakulasuriya et al., 2021; Tang et al., 2025). OSMF is driven by established carcinogenic exposure, chronic inflammation, tissue hypoxia, epithelial atrophy, and fibrotic matrix remodeling, and is therefore recognized as a high-risk OPMD (Warnakulasuriya et al., 2021; Tang et al., 2025; Hu Z-Y. et al., 2026; Xiong et al., 2025). Cohort studies have reported an approximately 5.1% malignant transformation rate for OSMF, with a median time to malignant transformation of approximately 36 months. However, substantial variability exists among studies, which may be attributable to differences in population characteristics, diagnostic criteria, histopathological grading, and follow-up duration (Villa et al., 2025).

Although OLK, PVL, OE, OLP, and OSMF differ markedly in their clinical manifestations and rates of malignant transformation, they share common biological features, including progressive disruption of epithelial homeostasis and remodeling of the surrounding microenvironment. Notably, malignant transformation of OPMDs does not follow a uniform linear pathway. Instead, distinct disease entities exhibit unique evolutionary patterns, in which chronic inflammation, oxidative stress, genetic and epigenetic alterations, immune dysregulation, epithelial-mesenchymal interactions, and microbiota dysbiosis contribute to varying extents. As shown in Figure 1, OPMDs do not represent a single precancerous entity but rather a highly heterogeneous spectrum of disorders. Different diseases progress along disease-specific evolutionary trajectories and may ultimately develop into OSCC. This conceptual framework highlights the heterogeneity of OPMDs and provides a basis for understanding the diverse molecular mechanisms underlying their progression.

**FIGURE 1:**
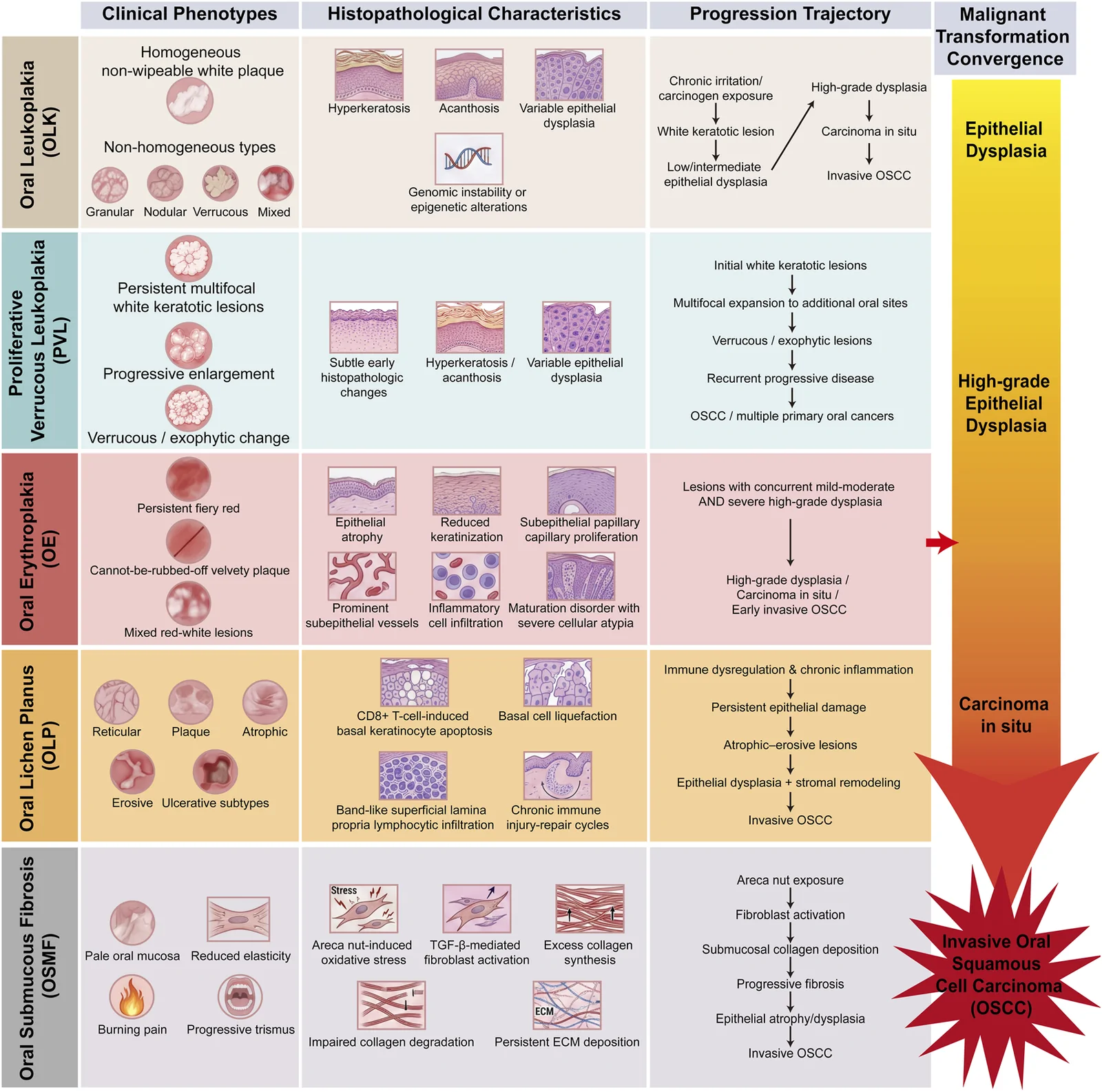
Clinical heterogeneity, histopathological characteristics, and malignant transformation trajectories of major oral potentially malignant disorders. Major OPMDs, including oral leukoplakia (OLK), proliferative verrucous leukoplakia (PVL), oral erythroplakia (OE), oral lichen planus (OLP), and oral submucous fibrosis (OSMF), exhibit distinct clinical phenotypes, histopathological features, and progression patterns. OLK is characterized by heterogeneous keratotic lesions and variable epithelial dysplasia; PVL by persistent multifocal progression, verrucous change, recurrence, and multiple primary cancers; OE by epithelial atrophy, marked atypia, and frequent high-grade dysplasia; OLP by chronic immune-mediated epithelial injury and stromal remodeling; and OSMF by areca nut-associated fibrosis and extracellular matrix accumulation. Despite these distinct trajectories, high-risk lesions may progress through epithelial dysplasia and carcinoma *in situ* to invasive oral squamous cell carcinoma (OSCC).

## 3 Disease-specific pathways underlying malignant transformation

### 3.1 Oral leukoplakia: genomic instability, immunosuppression, and field cancerization effects

OLK represents one of the most representative OPMDs characterized by concurrent clinical and molecular heterogeneity. Histological assessment of epithelial dysplasia remains the most widely used parameter for risk evaluation; however, dysplasia grading is limited by substantial subjectivity and poor interobserver agreement (Odell et al., 2021; Khoury et al., 2022). Accumulating evidence indicates that *TP53* mutations, *CDKN2A* inactivation, EGFR overexpression, Cyclin D1 upregulation, activation of the PI3K/AKT/β-catenin pathway, and loss of heterozygosity at 3p, 9p, and 17p loci contribute to malignant transformation of OLK (Wils et al., 2023; Normando et al., 2023; Ghosh et al., 2022; Monteiro et al., 2022; Jiang et al., 2001; Li et al., 2025a; Cai et al., 2023). The burden of copy number alterations and specific genomic changes in leukoplakia tissues, including 3p14 deletion, 8p23 deletion, and 20p11 amplification, are associated with increased malignant transformation risk (Wils et al., 2023; Ramos-García et al., 2022); *TP53* mutations also represent relatively robust predictors of malignant progression.

Beyond genetic alterations within epithelial cells, dysregulation of the OLK immune microenvironment is increasingly recognized as an early event during malignant transformation. A single-cell RNA-sequencing study of human tissue identified IDO1-positive macrophages as a potential immunosuppressive cell population associated with the malignant transformation of oral leukoplakia. *In vitro* and *in vivo* experiments further indicated that these cells can be activated through the IFN-γ–JAK–STAT pathway and interact with exhausted T cells, thereby facilitating immune evasion (Zhang Y. et al., 2024). Tobacco exposure may reprogram macrophage glutamine metabolism through the nicotine–α7nAChR axis, inducing M2 polarization and secretion of immunosuppressive mediators such as IL-10, thereby promoting dysregulated proliferation of oral epithelial cells (Zhu et al., 2021). Moreover, emerging evidence implicates several regulatory networks, including the migrasome–PTGES–PGE2–SPP1-positive macrophage signaling cascade, macrophage–BMP5 axis, FAP–ITGB1–PI3K/AKT pathway, HOXC-AS1/EZH2/HOXC9 axis, and DIRAS2–RAF/MEK/MAPK pathway, as potential mechanisms and therapeutic targets in OLK malignant transformation (Li et al., 2025a; Jiang et al., 2026; Li et al., 2026; Chen et al., 2025; Li et al., 2025b).

Field cancerization effects may explain why some patients experience recurrence, malignant transformation, or second primary tumors in adjacent or distant mucosal sites despite surgical excision of focal lesions. Low-depth whole-genome sequencing and morphology-associated copy number alteration (CNA) analysis have demonstrated that morphologically normal or low-grade dysplastic epithelial regions may already harbor aneuploidy or high-risk CNA clones, suggesting that molecular alterations may precede histopathological evidence of malignant transformation (Li et al., 2021).

### 3.2 Proliferative verrucous leukoplakia: multifocal progression, immune dysregulation, and field cancerization

PVL appears to follow a pattern of malignant progression distinct from the conventional model of focal, stepwise progression typically associated with oral leukoplakia (OLK). Its persistent multifocality, continued emergence of new lesions at anatomically distinct sites, high recurrence rate after local treatment, and pronounced propensity for multiple primary cancers are consistent with a field cancerization phenotype (Palaia et al., 2021). This conceptual framework provides a plausible explanation for why excision of a single lesion does not eliminate the subsequent risk of new lesions or malignant transformation elsewhere in the oral mucosa.

Immune remodeling may contribute to this biological behavior. PVL lesions exhibit increased infiltration of CD3^+^ and CD8^+^ T cells within the subepithelial compartment, with higher CD8^+^ T-cell density being associated with high-risk epithelial dysplasia (Fernandes et al., 2023). However, concurrent alterations in cytokine profiles and T-cell subset imbalance suggest that prominent lymphocytic infiltration does not necessarily translate into effective antitumor immunity. In parallel, increased numbers of CD163^+^ and FXIIIa^+^ macrophages, together with reduced numbers of mature dendritic cells, have been observed within PVL lesions, suggesting impaired antigen presentation and a shift toward a locally immunosuppressive microenvironment (Palaçon et al., 2023).

Multi-omics studies provide further evidence that PVL is biologically distinct from conventional oral leukoplakia. Comparative transcriptomic analyses have identified distinct gene expression patterns involving altered keratinization and stromal and extracellular matrix (ECM)-related signatures, while genome-wide DNA methylation studies have revealed widespread DNA hypermethylation and proteomic analyses have identified disease-associated protein expression profiles (Farah et al., 2024; Herreros-Pomares et al., 2021; Arroyo et al., 2023). Although these studies are limited by relatively small cohort sizes and require validation in independent longitudinal studies, their findings collectively suggest that PVL does not merely represent conventional OLK with a higher rate of malignant transformation, but rather a biologically distinct disease entity characterized by widespread epithelial alterations, persistent molecular remodeling, and dysregulated immune–stromal surveillance.

### 3.3 Oral erythroplakia: a high-risk phenotype driven by high-grade dysplasia

Compared with OLK, molecular studies of OE remain relatively limited; however, its clinicopathological risk profile is more clearly defined and concentrated. Erythroplakic mucosa commonly exhibits epithelial thinning, impaired maturation, marked cytological atypia, and vascular dilation, resulting in a clinical red appearance that frequently corresponds to high-grade pathological alterations (Shafer and Waldron, 1975). OE can be regarded as a high-risk OPMD primarily driven by high-grade dysplasia, with malignant progression often occurring within a relatively short time frame.

Mechanistically, OE is characterized by a high frequency of *TP53* mutations, with mutation rates reported to reach approximately 46% (Qin et al., 1999). TP53 mutations have been detected across different histopathologic stages of oral erythroplakia (OE), suggesting that disruption of the p53 pathway may occur relatively early during disease progression. In addition, stem cell-associated epithelial phenotypes may contribute to malignant progression. ALDH1 expression has been associated with an increased risk of malignant transformation, while coexpression of ALDH1 and Bmi1 was associated with an approximately 8.6-fold higher transformation risk. Collectively, these findings suggest that OE progression may involve early tumor-suppressor disruption together with the acquisition of stem-like epithelial phenotypes (Qin et al., 1999; Feng et al., 2013).

Furthermore, OE exhibits prominent field cancerization features, with some patients developing multifocal or multiple OSCCs. Increased expression of cancer stem cell-associated markers, including podoplanin and ABCG2, further suggests that cancer stem-like cells may contribute to field cancerization processes (Yang et al., 2020). Therefore, OE should be considered a high-risk phenotype driven by high-grade dysplasia. Clinically, prompt biopsy with adequate tissue sampling is essential, and management strategies, including complete excision or close surveillance, should be guided by histopathological grade.

### 3.4 Oral lichen planus: an immune-mediated chronic pro-tumorigenic niche

The central pathogenic mechanism of OLP involves T cell-mediated chronic mucosal inflammation. CD8^+^ T cells target basal keratinocytes, inducing liquefaction degeneration of basal cells and repeated damage-repair cycles, thereby establishing a persistent inflammatory microenvironment (Louisy et al., 2024; El-Howati et al., 2023; Maciel et al., 2025). Inflammatory mediators and signaling pathways, including TNF-α, IFN-γ, IL-6, IL-17, NF-κB, STAT3, and COX-2, may promote cellular proliferation, resistance to apoptosis, and DNA damage accumulation (Maciel et al., 2025; Al-Mohaya et al., 2016; Keim-Del Pino et al., 2024; Yao et al., 2025; Zhou et al., 2026).

Single-cell and spatial omics studies have further delineated the immunopathological landscape of OLP. Activated CD8^+^ Trm/Tem cells, TCR/BCR clonal expansion, pro-inflammatory myeloid activation, and aberrant fibroblast–immune cell–epithelial cell interactions collectively shape the pathological foundation of OLP (Li Q. et al., 2023). SFRP2^+^Wnt5a^+^ fibroblasts may recruit immune cells through chemokines such as CXCL12 and CXCL14 and establish an inflammatory positive feedback loop with CD8^+^ T cells (Cheng et al., 2025). A small-scale single-cell RNA-sequencing study of human tissue identified increased infiltration of unconventional T cells in OLP lesions. These cells exhibited a stress-response phenotype characterized by increased heat shock protein expression, enhanced NF-κB activation, reduced cytotoxic function, and resistance to apoptosis, suggesting that this population may be involved in sustaining local inflammation (Zhao et al., 2024). Beyond T cell-mediated immunity, spatial transcriptomic analysis of OLP tissues identified tertiary lymphoid structure-associated B-cell maturation and plasma-cell differentiation. Retrospective immunohistochemical validation further showed that these features were associated with unfavorable clinical outcomes (Yang et al., 2025). PA28γ-high-expressing keratinocytes may induce dendritic cell maturation and promote CD4^+^ T cell differentiation toward the Th1 phenotype *via* CCL5-CD44 signaling (Wang et al., 2024). Spatial proteomic studies of tongue OLP have proposed a KRT17-mediated positive feedback model, in which basal KRT17 upregulation contributes to epithelial barrier disruption; damaged epithelium releases IL-1β and IL-6 to recruit T cells, while infiltrating immune cells further enhance KRT17 expression, creating a self-amplifying cycle of epithelial injury, inflammatory escalation, and further epithelial damage (Zhan et al., 2026).

Although the malignant transformation risk of OLP is relatively low, it is not negligible. This risk may be associated with persistent inflammation, tongue involvement, erosive or atrophic phenotypes, smoking and alcohol exposure, hepatitis C virus (HCV) infection, *TP53* mutations, abnormal KRT17/PI3 expression, and upregulation of pathogenic Th17-related genes (Louisy et al., 2024; González-Moles et al., 2022; Maciel et al., 2025; de Lanna et al., 2022). Therefore, OLP should not be simply classified as a highly malignant-potential precancerous lesion. Instead, in a subset of patients, chronic immune-mediated inflammation may establish a persistent pro-tumorigenic niche that facilitates malignant transformation.

### 3.5 Oral submucous fibrosis: a matrix-driven precancerous condition characterized by fibrotic remodeling

The major etiological exposure associated with OSMF is areca nut consumption, particularly its principal bioactive component, arecoline. Arecoline induces sustained activation of the TGF-β/SMAD pathway, promoting fibroblast activation and collagen deposition. An imbalance between MMPs and TIMPs inhibits collagen degradation, ultimately resulting in extracellular matrix stiffening and fibrous band formation (Hu Z-Y. et al., 2026; Xu J. et al., 2024; Tilakaratne et al., 2006). Progressive fibrosis leads to vascular compromise and tissue hypoxia, activating HIF-1α-dependent hypoxic responses that promote aberrant angiogenesis, epithelial–mesenchymal interactions, and selection of malignant phenotypes (Tang et al., 2025). In addition, epithelial atrophy compromises mucosal barrier integrity, facilitating penetration of exogenous carcinogens and further increasing malignant transformation risk.

Multi-omics studies suggest that low-dose arecoline exerts stage-dependent regulatory effects during OSMF and OSCC progression. In fibroblasts, arecoline primarily regulates Wnt signaling, integrin-related interactions, and extracellular matrix remodeling. In OSCC cells, however, it predominantly influences chromatin assembly, DNA replication, cell-cycle regulation, and epigenetic reprogramming (Li Z. et al., 2023).

Therefore, OSMF should not be regarded merely as a fibrotic lesion with malignant potential; rather, it represents a matrix-driven precancerous disorder in which remodeling of the epithelial–stromal niche facilitates malignant transformation.

Taken together, these disease-specific mechanisms highlight the marked mechanistic heterogeneity underlying malignant transformation across OPMDs. As summarized in Figure 2, the following section focuses on shared biological processes across lesion types that collectively contribute to progression toward oral squamous cell carcinoma.

**FIGURE 2:**
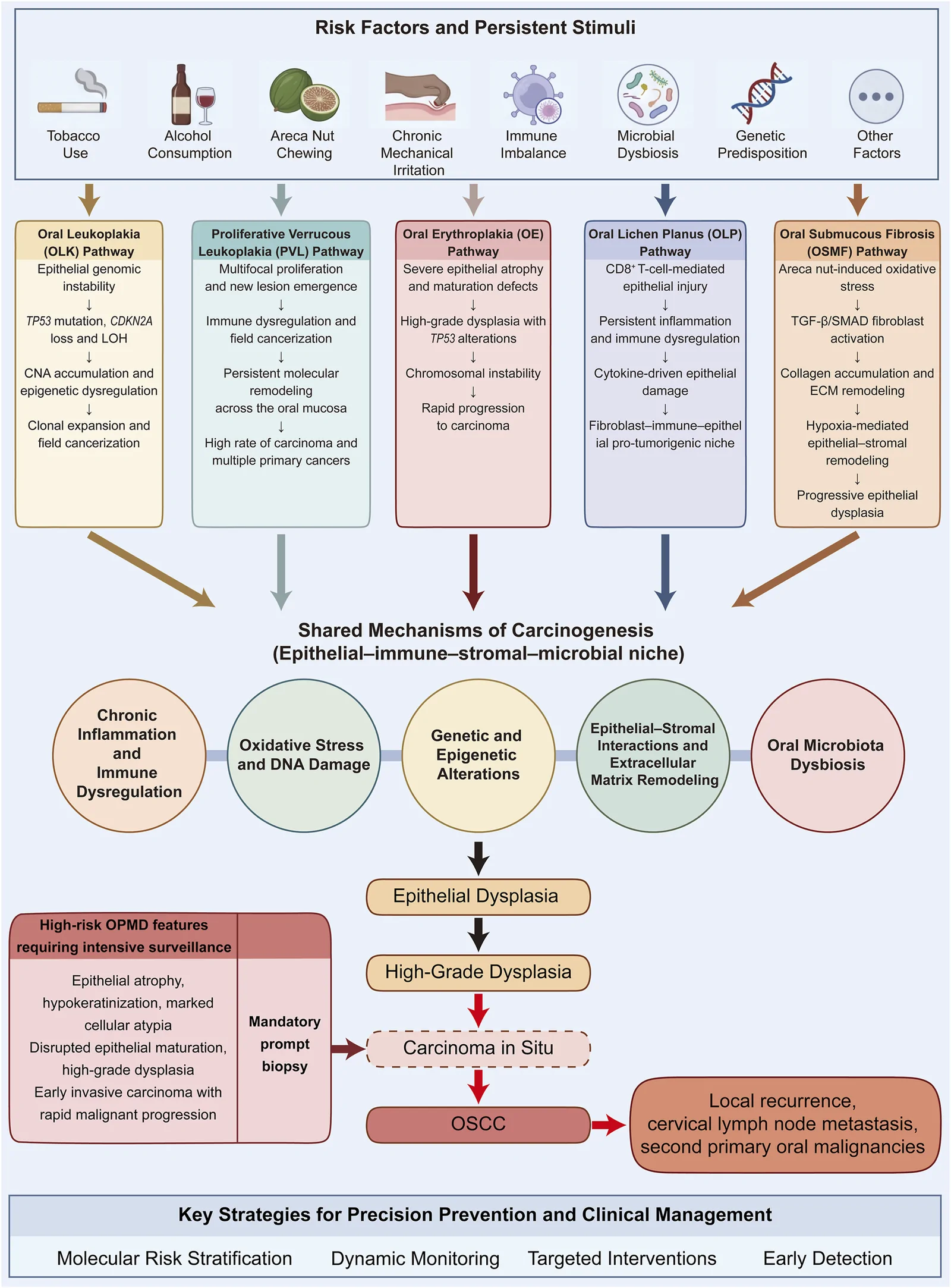
Integrated model of risk factors, disease-specific pathways, shared carcinogenic mechanisms, and clinical management strategies in OPMD progression to OSCC. Persistent risk factors and pathogenic stimuli contribute to the development and progression of major OPMDs, including OLK, PVL, OE, OLP, and OSMF, through distinct disease-specific pathways. Despite their heterogeneity, these disorders share carcinogenic mechanisms involving chronic inflammation and immune dysregulation, oxidative stress and DNA damage, genetic and epigenetic alterations, epithelial–stromal interactions, extracellular matrix remodeling, and oral microbiota dysbiosis. Progressive accumulation of these alterations may promote epithelial dysplasia, high-grade dysplasia, carcinoma *in situ*, and ultimately OSCC. High-risk features warrant intensive surveillance and prompt biopsy, while molecular risk stratification, dynamic monitoring, targeted interventions, and early detection support precision prevention and clinical management.

## 4 Common mechanisms underlying the progression of OPMDs to OSCC

### 4.1 Chronic inflammation and immune dysregulation

Chronic inflammation is a common feature across OPMDs and serves as a critical link between persistent stimuli, epithelial dysplasia, and malignant transformation. OLP is characterized by T cell-mediated immune inflammation, in which CD8^+^ T cells target basal keratinocytes, inducing basal cell injury and repetitive damage-repair cycles. OLK and OSMF also exhibit inflammatory cell infiltration, increased oxidative stress, and elevated inflammatory mediators, indicating that inflammation is not merely a secondary phenomenon but a fundamental component of precancerous microenvironment establishment (Tang et al., 2025; Maciel et al., 2025; Peng et al., 2023; Go et al., 2026). Inflammatory mediators and signaling pathways, including IL-6, TNF-α, IL-17, COX-2, NF-κB, and STAT3, promote dysregulated epithelial proliferation, resistance to apoptosis, angiogenesis, extracellular matrix degradation, and epithelial–mesenchymal transition (EMT). Meanwhile, persistent ROS/RNS production contributes to DNA damage accumulation and genomic instability, thereby facilitating malignant transformation (González-Moles et al., 2022; Normando et al., 2023; Maciel et al., 2025; Keim-Del Pino et al., 2024; Husein-ElAhmed and Steinhoff, 2022; Kim et al., 2025).

Recent studies further indicate that OPMD progression is not merely characterized by enhanced inflammation but involves a transition from immune surveillance toward immunosuppression. Immunosuppressive populations, including regulatory T cells (Tregs), myeloid-derived suppressor cells (MDSCs), M2 macrophages, and cancer-associated fibroblasts (CAFs), suppress effector T-cell function through mediators such as IL-10, TGF-β, VEGF, and MMPs, thereby promoting angiogenesis and tissue remodeling. Furthermore, upregulation of immune checkpoints, including PD-1/PD-L1, CTLA-4, and TIM-3, suggests that some OPMDs may already exhibit early immune evasion characteristics during the precancerous stage (Zhu et al., 2021; Tang et al., 2025; Ghosh et al., 2022; Li et al., 2024; Yokomizo et al., 2025; Deng S. et al., 2022). Single-cell studies have revealed that IDO1+ macrophages are enriched during OLK malignant transformation at the precancerous stage. Activated through the IFN-γ–JAK–STAT pathway, these macrophages interact with exhausted T cells to promote the establishment of an immunosuppressive microenvironment (Zhang Y. et al., 2024). Similar immune dysregulation has also been observed in PVL, with alterations in T-cell subsets accompanied by changes in macrophage and dendritic-cell composition, suggesting that prominent immune-cell infiltration does not necessarily translate into effective immune surveillance (Fernandes et al., 2023; Palaçon et al., 2023). Multiplex immunofluorescence studies have also demonstrated increased expression of IL-23R, CD68, and CD163, together with an elevated CD163/CD11c ratio, in OLK and OLP, suggesting that M2 macrophage polarization represents a shared mechanism underlying immune microenvironment remodeling in OPMDs (Trumet et al., 2025).

Therefore, chronic inflammation and immune dysregulation collectively shape the pro-tumorigenic niche of OPMDs and provide a rationale for therapeutic strategies targeting IL-23, IDO1, PD-1/PD-L1, and macrophage populations.

### 4.2 Oxidative stress and DNA damage

Tobacco, alcohol, areca nut exposure, and chronic inflammation can induce persistent generation of reactive oxygen/nitrogen species (ROS/RNS), placing the oral mucosa in a pro-oxidative state. Excessive ROS can induce DNA single- and double-strand breaks, oxidative base lesions such as 8-OHdG, lipid peroxidation, and mitochondrial dysfunction. Through activation of pathways including NF-κB, STAT3, PI3K/AKT, and HIF-1α, ROS promote dysregulated proliferation, resistance to apoptosis, and inflammatory amplification, thereby providing a basis for mutation accumulation and clonal selection in epithelial cells (Pras et al., 2017; Saso et al., 2022; Qin et al., 2023; Reuter et al., 2010; Wiśniewski et al., 2026).

In OLK, salivary redox studies have demonstrated increased levels of lipid peroxidation products, including MDA and TBARS, accompanied by decreased antioxidant defense factors such as GSH, uric acid, vitamins C and E, and GST, indicating the coexistence of oxidative damage and antioxidant depletion. Although 8-OHdG demonstrates only an increasing trend in some studies, its expression appears to increase with the severity of epithelial dysplasia, suggesting that oxidative DNA damage may contribute to OLK progression (Wiśniewski et al., 2026). In OSMF, arecoline induces ROS production and activates profibrotic pathways, including TGF-β, JNK, and ATF2, promoting fibroblast activation, collagen deposition, and tissue hypoxia. Elevated levels of MDA, LPO, 8-OHdG, NO, and serum copper, together with decreased antioxidant parameters such as SOD, GPx, and vitamins C and E, are associated with disease stage and histopathological severity (Saso et al., 2022; Pant et al., 2016; Khanna et al., 2013). In OLP, oxidative stress is closely intertwined with immunometabolic dysregulation. Keratinocyte pyroptosis, mitochondrial damage, mtDNA release, and activation of the cfDNA-cGAS-STING pathway may further induce IL-1β, NF-κB, and interferon-related inflammatory responses, establishing a positive feedback loop of “cell injury–DNA release–innate immune activation–inflammatory amplification” (Hu Y. et al., 2026; Deng J. et al., 2022; Wei et al., 2026).

Therefore, oxidative stress is not merely a secondary feature of OPMDs but represents a critical mechanism linking exogenous exposure, chronic inflammation, DNA damage, and malignant progression. However, oxidative stress-related biomarkers still require standardized assessment and prospective validation before their clinical application.

### 4.3 Genetic and epigenetic alterations

Genetic and epigenetic alterations constitute the fundamental molecular basis underlying the progression of OPMDs to OSCC. Alterations in *TP53*, *CDKN2A*, EGFR, Cyclin D1, and PI3K/AKT/mTOR signaling pathways contribute to cell-cycle dysregulation, evasion of apoptosis, aberrant proliferation, and genomic instability; however, their frequencies and biological significance vary among distinct OPMDs (González-Moles et al., 2022; Tsui et al., 2009; Prime et al., 2021; Cabral et al., 2025; Ramos-García et al., 2018).

In OLK, *TP53* mutations, *CDKN2A* inactivation, EGFR overexpression, Cyclin D1 upregulation, and loss of heterozygosity (LOH) at loci including 3p, 9p, and 17p are associated with increased malignant transformation risk (Kumari et al., 2022; Wils et al., 2023; Kobayashi et al., 2013). Increased copy number alteration (CNA) burden and specific genomic alterations, including 3p14 deletion, 8p23 deletion, and 20p11 amplification, may better reflect molecular high-risk states than conventional histopathological grading (Wils et al., 2023). LOH studies further suggest that coordinated disruption of tumor suppressor pathways, including 17p/*TP53*, 9p/*CDKN2A*, 3p/*FHIT*, 13q/Rb, 5q/*APC*, and 10q/*PTEN*, represents a major genetic mechanism involved in oral carcinogenesis (Gerstung et al., 2020; Rangel et al., 2022).

Epigenetic alterations may precede morphological malignant transformation and include abnormal DNA methylation, histone modifications, and dysregulation of non-coding RNAs. Evidence of early epigenetic remodeling has also been reported in proliferative verrucous leukoplakia (PVL). A small-scale genome-wide DNA methylation study revealed widespread hypermethylation and identified several candidate genes showing methylation alterations concordant with those observed in oral squamous cell carcinoma (OSCC), suggesting that DNA methylation may be involved in the early progression of PVL. However, the mechanistic significance and predictive value of these methylation alterations require further validation in larger longitudinal cohorts (Herreros-Pomares et al., 2021). In OLK, alterations in miRNA expression profiles are relatively well established, with miR-21, miR-31, miR-184, and miR-181b frequently upregulated, whereas miR-9 and miR-1 are downregulated. Among these, miR-375 demonstrates favorable discriminative performance between progressive and non-progressive OPMDs, while miR-31 shows consistent independent predictive value (Harrandah et al., 2016; Hung et al., 2016; Kaunein et al., 2021). Long non-coding RNAs (lncRNAs) and circular RNAs (circRNAs) also contribute to OLK malignant transformation. For example, *HOXC-AS1* competitively binds *EZH2* to relieve transcriptional repression of *HOXC9*, thereby promoting cancer stemness and epithelial–mesenchymal transition (EMT) (Chen et al., 2025). In OLP, alterations involving miR-31, miR-181a, circRNAs, the cfDNA-STING axis, and GSK-3β-FTO-VDR-mediated m6A regulation highlight the close interplay between epigenetic regulation and immune-inflammatory responses, which may collectively sustain chronic inflammation and epithelial dysfunction (Wei et al., 2026; Zhang J. et al., 2023; Fan et al., 2025; Huang et al., 2021).

Overall, integrated assessment of multilayer molecular alterations may provide a more effective strategy for OPMD risk stratification than reliance on single biomarkers.

### 4.4 Epithelial–stromal interactions and extracellular matrix remodeling

Carcinogenesis is not an isolated event occurring within epithelial cells but rather a process of ecological niche remodeling involving epithelial cells, fibroblasts, endothelial cells, immune cells, and the extracellular matrix. Basement membrane disruption, EMT activation, MMP upregulation, VEGF-mediated aberrant angiogenesis, fibroblast activation, and acquisition of CAF-like phenotypes collectively promote the transition from dysplasia to invasive carcinoma (Kumari et al., 2022; Ghosh et al., 2022; Zhang Y. et al., 2024; Pandiar et al., 2023; Correa-Fernández et al., 2025).

OSMF represents a prototypical example of carcinogenesis driven by a fibrotic microenvironment. Arecoline promotes myofibroblast differentiation, excessive collagen deposition, and tissue hypoxia through mechanisms involving TGF-β/SMAD signaling, ROS accumulation, HIF-1α activation, and MMP/TIMP imbalance. The stiffened matrix further enhances epithelial proliferation, migration, and invasion through YAP/TAZ, PI3K/AKT, and EMT-related signaling, establishing a continuous “fibrosis–hypoxia–EMT–malignant transformation” cascade (Hu Z-Y. et al., 2026; Xu J. et al., 2024; Liao et al., 2024; T et al., 2008; Li et al., 2019; Shetty et al., 2025). Recent studies indicate that stromal cells are not merely passive responders but may actively drive OPMD progression. In OLK, FAP interacts with ITGB1 to activate the PI3K/AKT signaling axis, promoting oral epithelial cell proliferation and migration, whereas suppression of FAP expression may delay malignant transformation of OLK (Li et al., 2025a). OLK-associated stromal fibroblast transcription factors, including SOX11 and GLIS2, are associated with EMT, angiogenesis, and poor prognosis, suggesting that stromal reprogramming may precede invasive carcinoma formation (Li Y. et al., 2025). PVL may also exhibit a distinct pattern of stromal remodeling. Transcriptomic analyses have identified alterations associated with cell motility and extracellular matrix (ECM) remodeling. Direct comparison with conventional oral leukoplakia (OLK) revealed relative downregulation of connective tissue- and ECM-related signatures, together with an inferred reduction in fibroblast abundance. These findings suggest that PVL may exhibit a distinct epithelial–stromal state rather than a uniformly activated fibrotic phenotype; however, these observations require further validation at the cellular level (Farah et al., 2024; Llorens et al., 2021). In OSMF, MMP9, SPARC, and ITGA5 have been implicated in driving the transition from fibrosis-associated type 2 EMT to tumor invasion-associated type 3 EMT. Furthermore, THBS1-mediated inhibition of angiogenesis, COMP-mediated abnormal assembly of type I collagen, and CAF-like cell formation can collectively alter tissue mechanics and the immune microenvironment (Xiong et al., 2025; Xu J. et al., 2024; Shetty et al., 2025; Yang X. et al., 2024).

Therefore, malignant transformation of OPMDs should be understood as the result of co-evolution between abnormal epithelial clones and a pro-tumorigenic stromal niche. Therapeutic targeting of FAP, TGF-β, MMP9, ITGA5, YAP/TAZ, or angiogenic pathways may represent promising strategies for interrupting precancerous progression.

### 4.5 Oral microbiota dysbiosis

Oral microbiota dysbiosis contributes to OPMD progression toward OSCC through mechanisms involving persistent inflammation, epithelial barrier disruption, DNA damage induction, immune modulation, and promotion of EMT. Microorganisms that have been extensively investigated include *Fusobacterium* nucleatum, Porphyromonas gingivalis, and *Candida* albicans (Kim et al., 2025; Gurizzan et al., 2025; Beibei et al., 2024).

F. nucleatum is enriched in OSCC and certain high-risk OPMDs and can activate signaling pathways including NF-κB, STAT3, TLR/MyD88, and integrin/FAK, thereby promoting inflammation, epithelial proliferation, EMT, and invasion. Its metabolites, including hydrogen sulfide, can further induce oxidative stress and DNA damage (Santacroce et al., 2025; Soghli et al., 2025; Wu et al., 2026). Increased abundance and functional activity of F. nucleatum in hypoxic OPMDs suggest that hypoxia, inflammation, and microbiota dysbiosis may collectively establish a high-risk pro-tumorigenic niche (Gurizzan et al., 2025). P. gingivalis, a key periodontal pathogen, activates signaling pathways such as ERK1/2-Ets1, p38/HSP27, and PAR2/NF-κB through virulence factors including lipopolysaccharide and gingipains. These pathways increase IL-6, IL-8, TNF-α, and MMP expression, thereby promoting chronic inflammation, extracellular matrix degradation, epithelial proliferation, and tumor invasion (Wu et al., 2026; Urzică et al., 2025). Emerging data also suggest a potential association between the oral microbiome and proliferative verrucous leukoplakia (PVL). A matched case-control study reported an increased overall burden of red- and orange-complex periodontal pathogens in patients with PVL, including *Fusobacterium* nucleatum, Porphyromonas gingivalis, Tannerella forsythia, and *Treponema* denticola. However, given the retrospective design of the study, these findings do not establish a causal relationship between microbial dysbiosis and malignant transformation (Liska et al., 2025). Among fungal microbiome alterations, C. albicans has been closely associated with non-homogeneous leukoplakia and OLP (Beibei et al., 2024; Gupta et al., 2021). *Candida* can disrupt mucosal barriers through adhesion, hyphal invasion, and secretion of hydrolytic enzymes. Elevated levels of pro-inflammatory cytokines, including IL-6, IL-8, IL-17, and TNF-α, in Candida-associated oral leukoplakia contribute to the establishment of a pro-carcinogenic inflammatory microenvironment (Gupta et al., 2021). Certain strains can metabolize ethanol into acetaldehyde, generating DNA adducts and enhancing tobacco- and alcohol-associated carcinogenic effects (Wu et al., 2026; Marttila et al., 2013). Antifungal treatment reduces pro-inflammatory cytokine levels in patients with Candida-positive leukoplakia, suggesting that *Candida* may contribute to lesion progression (Gupta et al., 2021).

Overall, oral microbiota dysbiosis represents an important microenvironmental factor contributing to OPMD malignant transformation; however, causal relationships require confirmation through longitudinal studies.

## 5 Biomarkers for malignant transformation risk prediction and clinical stratification

Current OPMD biomarkers can be broadly classified into five categories: clinical, pathological, molecular, immune-stromal, and fluid-based biomarkers (see Table 2). Clinically, heterogeneous lesions, mixed red-white lesions, multifocal lesions, progressive lesions, involvement of the ventral tongue or floor of the mouth, long-term tobacco/alcohol/areca nut exposure, and worsening trismus in OSMF are associated with increased malignant transformation risk (Pimenta-Barros et al., 2025; Kumari et al., 2022; Maciel et al., 2025).

**TABLE 2:** Current biomarkers for predicting malignant transformation risk in oral potentially malignant disorders.

Biomarker category	Representative biomarkers/features	Associated OPMDs	Biological significance	Clinical value and limitations	Representative references
Clinical	Non-homogeneous/mixed red-white lesions; persistent/recurrent multifocal or progressive/new lesions; tongue/floor-of-mouth involvement; tobacco/alcohol/areca exposure; progressive trismus	OLK, PVL, OE, OLP, OSMF	Lesion phenotype, site-related risk, and exposure burden	Routinely accessible, but subjective and insufficient for individual prediction	(Pimenta-Barros et al., 2025; Thompson et al., 2021; Palaia et al., 2021; Lorenzo-Pouso et al., 2022; González-Moles et al., 2019; Tang et al., 2025)
Histopathological	OED grade; basement membrane disruption; Ki-67, p53, p16, podoplanin (pooled RR = 3.64), SOX2, ALDH1, Bmi1	Mainly OLK/OE	Proliferation, genomic instability, stemness, and malignant potential	OED is routinely used; IHC markers remain adjunctive and incompletely validated	(Odell et al., 2021; Monteiro et al., 2022; Cai et al., 2023; Feng et al., 2013; Correa-Fernández et al., 2025; de Vicente et al., 2019; Liu et al., 2013)
Genomic	TP53 mutation; CDKN2A alteration; 3p/9p/17p LOH; CNA risk score (AUC = 0.879; external AUC = 0.836–0.876); chromosomal instability; 9p21.3 loss	OLK, PVL, OE	Early genomic evolution and clonal selection	CNA models may improve prediction beyond conventional grading; prospective validation is needed	(Li et al., 2021; Wils et al., 2023; Cai et al., 2023; Qin et al., 1999; Prime et al., 2021; Cai et al., 2024; Hanna et al., 2024)
Epigenetic	DNA methylation; GPX3, DLG2, GATA3, HOXD10; miR-21/31/184/375; lncRNA HOXC-AS1; circRNAs	OLK, OLP, PVL	Proliferation, EMT, inflammation, and stemness	Promising but largely exploratory; prospective validation is needed	(Chen et al., 2025; Herreros-Pomares et al., 2021; Harrandah et al., 2016; Hung et al., 2016; Kaunein et al., 2021; Zhang et al., 2023a; Huang et al., 2021; Xu et al., 2021)
Immune microenvironment	IDO1+ macrophages; CD163/CD11c ratio; PD-1/PD-L1; IL-23R; CD8^+^ T-cell signatures; CD163^+^/FXIIIa^+^ macrophages; dendritic-cell alterations; KRT17-associated interactions	OLK, PVL, OLP	Immune escape and inflammatory remodeling	Emerging; longitudinal predictive validation remains limited	(Zhang et al., 2024a; Fernandes et al., 2023; Palaçon et al., 2023; Zhan et al., 2026; Deng et al., 2022a; Trumet et al., 2025)
Stromal/ECM	FAP, SOX11, GLIS2, MMP9, SPARC, ITGA5	OLK, OSMF	Fibroblast activation, ECM remodeling, and EMT	Emerging; further validation is required	(Li et al., 2025a; Shetty et al., 2025; Li et al., 2025c)
Fluid	Salivary IL-6/IL-8/TNF-α, cfDNA, miRNAs; podoplanin (AUC = 0.89; Se = 85%; Sp = 83%); methylation panels; Raman signatures (Se = 97%; Sp = 100%; n = 12)	OLK, OSCC, OPMDs	Non-invasive molecular detection	High translational potential; multicenter prospective validation is needed	(Liew et al., 2024; Uzun et al., 2026; Bastías et al., 2024; Sekaran and Ganapathy, 2022; Behl et al., 2025)

Pathologically, epithelial dysplasia grading, basement membrane integrity, inflammatory infiltration patterns, and markers such as Ki-67, p53, p16, podoplanin, SOX2, and ALDH1 may aid risk assessment; however, interobserver variability and limited reliability of single biomarkers restrict their clinical implementation (Monteiro et al., 2022; Cai et al., 2023; de Vicente et al., 2019; Liu et al., 2013; Zhang et al., 2017; You et al., 2023).

In OLK, *TP53* mutations, 3p/9p/17p LOH, CNA burden, *CDKN2A* inactivation, EGFR and Cyclin D1 alterations, DNA methylation profiles, and miRNA/lncRNA/circRNA panels represent some of the most actively investigated biomarker candidates (Li et al., 2021; Normando et al., 2023; Cai et al., 2023; Ramos-García et al., 2022; Prime et al., 2021; Kaunein et al., 2021; Graveland et al., 2013; Liew et al., 2024; Xu et al., 2021). Podoplanin, DcR2, DEC1, Ki-67, CD44v6, p73, HSP70-2, MUC1/MUC4, SOX11, and GLIS2 have also been proposed as candidate biomarkers for malignant transformation risk prediction or early detection (Monteiro et al., 2022; Go et al., 2026; Li Y. et al., 2025; Uzun et al., 2026; de Villalaín et al., 2023; Li et al., 2025d; Rathee et al., 2021). Among these approaches, whole-genome CNA scores, morphology-associated CNA analysis, and AI-based prediction models incorporating 9p deletion have demonstrated potential advantages over conventional pathological grading (Li et al., 2021; Cai et al., 2024; Cai et al., 2025). Notably, CNA-based risk prediction currently has relatively stronger longitudinal evidence. In a multicenter retrospective cohort of 431 patients with long-term follow-up, a whole-genome CNA score showed predictive performance in both internal and external validation cohorts; however, prospective validation is still required before routine clinical implementation (Cai et al., 2024). In contrast, research on molecular biomarkers for PVL remains relatively limited. Available evidence indicates that copy-number loss at 9p21.3 is associated with subsequent progression to OSCC among patients with high-risk PVL, whereas a genome-wide DNA methylation study identified several candidate epigenetic biomarkers, including GPX3, DLG2, GATA3, and HOXD10. However, the predictive value of these candidate biomarkers remains to be validated in larger longitudinal cohorts (Herreros-Pomares et al., 2021; Hanna et al., 2024). Risk prediction based on immune-related and stromal biomarkers is more complex, particularly in OLP. Given its relatively low overall malignant transformation rate, biomarker studies in OLP should avoid simply equating inflammatory activity with malignant potential. Tongue involvement, erosive/atrophic phenotypes, tobacco and alcohol exposure, HCV infection, erythematous lesions, *TP53* mutations, and alterations in KRT17, PI3, HIF1A, and IL1B may indicate increased malignant transformation risk (González-Moles et al., 2022; Maciel et al., 2025; Keim-Del Pino et al., 2024; de Lanna et al., 2022). Furthermore, cfDNA, miR-31/miR-181a, circRNA, spatial KRT17 expression patterns, and digital pathology features may serve as indicators of disease activity and treatment response (Zhan et al., 2026; Wei et al., 2026; Zhang J. et al., 2023; Huang et al., 2021).

Salivary biomarkers possess substantial translational potential owing to their non-invasive nature and feasibility for repeated sampling. IL-6, IL-8, TNF-α, miRNAs, circulating free DNA, PDPN, P90, methylation-based gene panels, and Raman spectroscopy signatures have been investigated for early detection of OLK and OSCC (Liew et al., 2024; Uzun et al., 2026; Bastías et al., 2024; Sekaran and Ganapathy, 2022; Wan et al., 2024; Behl et al., 2025). However, variations in sample processing, analytical platforms, and diagnostic thresholds among studies highlight the need for large-scale, multicenter, prospective validation.

## 6 Risk stratification and follow-up strategies

Reliance solely on histopathological grading to determine follow-up strategies is insufficient to meet the requirements of precision management. A more appropriate approach is to integrate clinical phenotypes, histopathological features, molecular alterations, immune microenvironmental characteristics, microbiological profiles, and exposure history to establish a dynamic risk stratification framework, with risk levels continuously updated according to lesion evolution during follow-up (Zhu et al., 2021; Hu Z-Y. et al., 2026; Cai et al., 2024; Li et al., 2025e; Adeoye et al., 2024; Lan et al., 2023) (see Table 3).

**TABLE 3:** Dynamic risk stratification and clinical management strategies for oral potentially malignant disorders.

Risk category	Representative lesions/features	Molecular/pathological characteristics	Suggested management
Low-risk OPMDs	Homogeneous OLK; stable reticular OLP; small lesions without high-risk anatomical sites	No or mild dysplasia; absence of high-risk molecular alterations	Risk-factor modification; routine surveillance; oral hygiene management
Intermediate-risk OPMDs	Non-homogeneous OLK; erosive/atrophic OLP; progressive OSMF; mild-to-moderate dysplasia	Partial molecular alterations; inflammatory activation; uncertain progression potential	Shortened surveillance intervals; repeat biopsy; imaging, salivary biomarkers, or molecular testing when appropriate
High-risk OPMDs	OE; PVL; severe dysplasia; carcinoma *in situ*; rapidly progressing multifocal lesions; OSMF with progressive trismus	TP53 alterations; high CNA burden; severe epithelial atypia; immune/stromal remodeling	Active intervention, including excision, laser ablation, PDT, and intensified surveillance
Very high-risk lesions	High-grade dysplasia with rapid progression; early invasive carcinoma; multiple high-risk features	Severe genomic instability; field cancerization; immune escape signatures	Immediate biopsy/excision and multidisciplinary management

Low-risk lesions generally include clinically stable lesions with limited size, absence of high-risk anatomical sites, absent or mild dysplasia, and no high-risk molecular features. Examples include some homogeneous leukoplakia and stable reticular OLP, for which routine surveillance, behavioral modification, and oral hygiene management are appropriate (Pimenta-Barros et al., 2025; Louisy et al., 2024; Gates et al., 2025; Duś-Ilnicka et al., 2026). Intermediate-risk lesions include non-homogeneous leukoplakia, atrophic/erosive OLP, progressive OSMF, mild-to-moderate dysplasia, or lesions harboring partial molecular high-risk features. These patients require shorter surveillance intervals, repeat biopsy when indicated, and integration of imaging, salivary biomarkers, or molecular testing to improve risk assessment (Pimenta-Barros et al., 2025; Tang et al., 2025; Gates et al., 2025). High-risk lesions include OE, proliferative verrucous leukoplakia, severe dysplasia or carcinoma *in situ*, rapidly progressing multifocal lesions, lesions with high-risk CNA or *TP53* alterations, and OSMF with progressive trismus. These patients are more likely to benefit from active intervention, including surgical excision, laser therapy, photodynamic therapy, or intensified surveillance (Tan et al., 2023; Li et al., 2021; Palaia et al., 2021; Das et al., 2022; Duś-Ilnicka et al., 2026; Tenore et al., 2025).

PVL should be regarded as a high-risk oral potentially malignant disorder (OPMD) irrespective of the epithelial dysplasia grade observed in a single biopsy (Thompson et al., 2021). Risk assessment should emphasize longitudinal clinicopathologic evolution, including progressive multifocal involvement, lesion enlargement, the emergence of new lesions, recurrence, and the development of erythema, nodular or verrucous thickening, ulceration, or changes in consistency; such changes should prompt repeat biopsy or multisite sampling (Thompson et al., 2021; Palaia et al., 2021; González-Moles et al., 2021). The prognostic value of epithelial dysplasia severity remains uncertain. In a longitudinal cohort of 36 patients, the degree of epithelial dysplasia was not significantly associated with overall malignant transformation, highlighting the limitations of relying on a single histopathologic assessment (Zhang C. et al., 2024). A previous history of oral squamous cell carcinoma (OSCC) and potential molecular features such as 9p21.3 loss may provide additional information for risk stratification, although their prognostic value requires further prospective validation (Hanna et al., 2024).

Digital pathology and artificial intelligence (AI) are transforming risk stratification approaches for OPMDs. A retrospective model-development study with external validation showed that OMMT-PredNet could identify epithelial dysplasia in OLK and predict malignant transformation risk without the need for manual region-of-interest (ROI) annotation. Similarly, a multicenter retrospective model-development and validation study showed that a pathomics-based model could predict 9p loss from routine hematoxylin and eosin (H&E)-stained sections. OLPNet, evaluated on an image-classification dataset and an external clinical dataset, likewise showed potential for smartphone image-based leukoplakia screening in resource-limited settings (Cai et al., 2025; Li et al., 2025e; Feng et al., 2026). However, current AI models remain limited by insufficient sample sizes, inconsistent annotation standards, lack of external validation, and inadequate follow-up endpoints. Therefore, they should currently be considered decision-support tools rather than independent systems capable of replacing clinical judgment.

## 7 Therapeutic and preventive strategies

Elimination of carcinogenic exposures represents the foundation of OPMD management. Smoking cessation, alcohol abstinence, and cessation of areca nut chewing not only contribute to lesion improvement but may also reduce recurrence and subsequent malignant transformation risk, particularly in OSMF (Louisy et al., 2024; Tang et al., 2025; Gates et al., 2025). For high-risk OLK, OE, and lesions with high-grade dysplasia, surgical excision or laser ablation remains the most direct approach for local disease control. However, excision cannot completely eliminate the field cancerization effect, making long-term postoperative surveillance essential (Bhattarai et al., 2024; Gates et al., 2025; Brignardello-Petersen, 2020).

In OLK treatment, laser therapy, photodynamic therapy (PDT), and local drug delivery systems have emerged as important therapeutic approaches in recent years. Meta-analyses suggest that, for homogeneous leukoplakia, Er,Cr:YSGG laser, Er:YAG laser, and CO_2_ laser combined with PDT may reduce recurrence rates and improve patient tolerability (Luo et al., 2024). A systematic review and meta-analysis based predominantly on single-arm clinical trials suggested that ALA-PDT may achieve favorable clinical responses in patients with homogeneous oral leukoplakia without dysplasia or with mild-to-moderate dysplasia, whereas its efficacy appeared to be more limited in those with severe dysplasia (Zhang R. et al., 2023). Emerging strategies include 5-aminolevulinic acid (ALA)-loaded adhesive hydrogels, organic semiconductor nanomaterial-mediated photodynamic/photothermal therapy, sonodynamic immunotherapy, rapamycin-loaded PVA-DOPG hydrogels, G-TA@Mn^2+^ hydrogels, low-molecular-weight fucoidan nanofiber membranes, cucurbitacin B, baicalein, black raspberry-based chemopreventive approaches, and topical imiquimod. However, the supporting evidence varies substantially across these approaches: most biomaterial- and pharmacological therapies have been evaluated only in preclinical models, ALA-loaded adhesive hydrogels have undergone preliminary evaluation in humans, whereas clinical evidence for topical imiquimod remains limited and is derived largely from small studies conducted under heterogeneous study settings (Lin et al., 2022; Lin et al., 2026; Xu et al., 2026; Du et al., 2024; Shi et al., 2024; Xu M. et al., 2024; Yang M. et al., 2024; Hou et al., 2022; Lança et al., 2025; Scharner et al., 2026).

PVL should prioritize local control of clinically suspicious lesions while recognizing that local treatment does not eliminate the field-wide risk across the oral mucosa. Surgery, laser ablation, and photodynamic therapy may provide local disease control; however, recurrence remains common, and there is currently insufficient evidence that these interventions alter the long-term natural history of PVL. Long-term surveillance of the entire oral mucosa is therefore essential (Zhang C. et al., 2024; Upadhyaya et al., 2018). Immunoprevention remains investigational. In a nonrandomized phase II study of 33 patients with high-risk PVL, nivolumab achieved an overall response rate of 36% and a 2-year cancer-free survival rate of 73%. Nevertheless, 27% of patients progressed to oral squamous cell carcinoma (OSCC), and approximately 21% experienced grade 3–4 immune-related adverse events. Pretreatment copy-number loss at 9p21.3 was associated with subsequent malignant transformation (Hanna et al., 2024). These findings support further biomarker-stratified clinical trials but remain insufficient to support the routine use of immune checkpoint inhibitors for the prevention of malignant transformation in PVL.

The primary goals of OLP treatment are to alleviate pain, burning sensations, and inflammatory activity, reduce acute exacerbations, and improve quality of life rather than achieving complete lesion resolution (Louisy et al., 2024). Topical super-potent corticosteroids remain the first-line therapy, whereas calcineurin inhibitors, including tacrolimus and pimecrolimus, are commonly used as second-line topical treatments (Utz et al., 2022; Deng X. et al., 2022). For moderate-to-severe, extensive, or refractory OLP, systemic therapies such as hydroxychloroquine, methotrexate, systemic corticosteroids, retinoids, azathioprine, mycophenolate mofetil, and thalidomide may be considered (Louisy et al., 2024; Vinay et al., 2024; Platais et al., 2023). Novel targeted therapies, including JAK inhibitors, apremilast, and IL-17/IL-23 inhibitors, have demonstrated rapid clinical responses in refractory erosive OLP; however, their efficacy requires confirmation through high-quality clinical trials (Husein-ElAhmed and Steinhoff, 2022; Balestri et al., 2022; Perschy et al., 2022). Evidence from prospective clinical studies and systematic reviews/meta-analyses suggests that, despite heterogeneity in the available evidence, 5-aminolevulinic acid photodynamic therapy (5-ALA-PDT) and low-level laser therapy may represent alternative treatment options for oral lichen planus (Sulewska et al., 2025; Hoseinpour Jajarm et al., 2018). In addition, a randomized clinical trial showed that a mucosa-targeted drug delivery system could reduce systemic glucocorticoid requirements in patients with severe oral lichen planus (Elsayed et al., 2026).

Treatment of OSMF mainly focuses on cessation of areca nut exposure, anti-inflammatory, antioxidant, antifibrotic, and functional rehabilitation strategies. Current approaches include local injections, hyaluronidase therapy, antioxidant supplementation, mouth-opening exercises, and antifibrotic interventions; however, the overall quality of evidence remains limited (Tang et al., 2025). Future studies should further investigate targeted interventions targeting TGF-β/SMAD, HIF-1α, Wnt, Notch, epigenetic reprogramming, and arecoline-associated metabolic pathways (Xu J. et al., 2024; Xiao et al., 2026; Cai et al., 2026; Huang et al., 2022).

## 8 Challenges and future perspectives

Current research and clinical management of OPMDs continue to face three major challenges. First, disease definitions and diagnostic criteria remain insufficiently standardized. In particular, difficulties in distinguishing OLP from OLLs, together with variations in diagnostic criteria and staging systems across different OPMDs, directly affect estimates of malignant transformation risk and comparability across studies. Methodological differences across studies, together with variations in study populations, follow-up duration, and outcome definitions, may partly explain the heterogeneity in reported estimates of malignant transformation risk. Second, many biomarker studies remain limited to single-center, small-scale, retrospective investigations. The lack of multicenter prospective longitudinal validation has hindered their translation into routine clinical practice. Third, most current studies focus on abnormalities at individual biological levels and remain insufficient to explain the real-world complexity of heterogeneous evolution within the same disease entity and convergent carcinogenesis among distinct OPMDs.

Despite their considerable promise, the translation of emerging technologies into clinical practice remains challenging. Single-cell and spatial omics are resource-intensive and can be affected by variations in tissue processing, analytical platforms, and computational workflows, whereas AI, digital pathology, and liquid biopsy approaches require greater standardization and reproducibility across centers. High implementation costs, limited multicenter validation data, evolving regulatory requirements, and difficulties in integrating these technologies into existing clinical workflows—including oral examination, biopsy, histopathological assessment, and long-term follow-up—remain major barriers to widespread clinical adoption.

Future research should prioritize international multicenter collaboration and establish standardized prospective longitudinal cohorts, with harmonized documentation of clinical phenotypes, pathological grading, treatment, follow-up, and malignant transformation endpoints. Integration of these clinical data with imaging, digital pathology, multi-omics, microbiome, and liquid biopsy datasets may facilitate the development of validated dynamic risk prediction models (Prime et al., 2021; Gurizzan et al., 2025; Adeoye and Su, 2026; Walsh et al., 2021; Sathasivam et al., 2021; Saha et al., 2026). For AI-based approaches, external validation and model interpretability should be emphasized to support transparent risk-stratified surveillance, repeat biopsy, and individualized intervention. Before routine clinical implementation, emerging technologies require standardized workflows, cross-center reproducibility, prospective validation of clinical utility, and appropriate regulatory and quality-control assessment; clinical trial endpoints should likewise extend beyond lesion response to progression to high-grade epithelial dysplasia, OSCC development, recurrence, and second primary cancers.

## 9 Conclusion

OLK, PVL, OE, OLP, and OSMF should not be considered merely five independent categories of oral mucosal diseases, but rather distinct points along a continuum from chronic injury/inflammation to dysplasia, microenvironmental remodeling, and malignant transformation. These disorders share common pro-tumorigenic mechanisms, including chronic inflammation, oxidative stress, genetic and epigenetic dysregulation, epithelial–mesenchymal interactions, and microbial dysbiosis; however, their dominant pathogenic pathways differ substantially.

OLK is characterized predominantly by genomic instability, immune suppression, and the field cancerization effect. PVL is characterized by persistent multifocal progression, pronounced field cancerization, and a sustained propensity for malignant transformation, with a high risk of developing multiple primary oral cancers. OE is characterized by rapid progression driven by high-grade dysplasia. OLP is associated with a chronic pro-tumorigenic niche shaped by immune-mediated inflammation. OSMF represents a premalignant condition driven by fibrotic stromal remodeling.

Future OPMD management should transition from a static strategy based on periodic observation after lesion detection toward dynamic risk stratification and precision intervention guided by clinical, pathological, molecular, and microenvironmental features. This transition will represent a key direction for improving early prevention, detection, and intervention strategies for oral cancer.

## Glossary

5-ALA-PDT: 5-Aminolevulinic Acid Photodynamic Therapy
8-OHdG: 8-Hydroxy-2′-deoxyguanosine
AI: Artificial Intelligence
AKT: Protein Kinase B
ALA: 5-Aminolevulinic Acid
BCR: B-Cell Receptor
CAFs: Cancer-Associated Fibroblasts
cfDNA: Cell-Free DNA
circRNA: Circular RNA
CNA: Copy Number Alteration
COX-2: Cyclooxygenase-2
DNA: Deoxyribonucleic Acid
ECM: Extracellular Matrix
EGFR: Epidermal Growth Factor Receptor
EMT: Epithelial–Mesenchymal Transition
GSH: Glutathione
GST: Glutathione S-Transferase
H&E: Hematoxylin and Eosin
HCV: Hepatitis C Virus
HIF-1α: Hypoxia-Inducible Factor-1α
HIV: Human Immunodeficiency Virus
IFN-γ: Interferon-γ
IL: Interleukin
JAK: Janus Kinase
lncRNA: Long Non-Coding RNA
LOH: Loss of Heterozygosity
MDA: Malondialdehyde
MDSCs: Myeloid-Derived Suppressor Cells
miRNA: MicroRNA
MMPs: Matrix Metalloproteinases
mTOR: Mechanistic Target of Rapamycin
NF-κB: Nuclear Factor-κB
OE: Oral Erythroplakia
OLK: Oral Leukoplakia
OLLs: Oral Lichenoid Lesions
OLP: Oral Lichen Planus
OPMDs: Oral Potentially Malignant Disorders
OSCC: Oral Squamous Cell Carcinoma
OSMF: Oral Submucous Fibrosis
PD-1: Programmed Cell Death Protein 1
PD-L1: Programmed Death-Ligand 1
PDT: Photodynamic Therapy
PI3K: Phosphoinositide 3-Kinase
PVL: Proliferative Verrucous Leukoplakia
RNA: Ribonucleic Acid
RNS: Reactive Nitrogen Species
ROI: Region of Interest
ROS: Reactive Oxygen Species
STAT3: Signal Transducer and Activator of Transcription 3
STING: Stimulator of Interferon Genes
TBARS: Thiobarbituric Acid-Reactive Substances
TCR: T-Cell Receptor
TGF-β: Transforming Growth Factor-β
TIMPs: Tissue Inhibitors of Metalloproteinases
TNF-α: Tumor Necrosis Factor-α
Tregs: Regulatory T Cells
VEGF: Vascular Endothelial Growth Factor
